# Congenital hepatic hemangioma: an unusual case report of pulmonary hypertension

**DOI:** 10.1186/s12887-023-04096-w

**Published:** 2023-06-07

**Authors:** Qianqin Zhou, Juan Jiang, Yinquan Xu, Hanyan Qiu, Xiaoyan Wen, Shaoqi He, Qin Lv

**Affiliations:** Neonatal Intensive Care Unit, The Ningbo Women and Children’s Hospital, 339 Liuting Rd, 315012 Ningbo, Zhejiang People’s Republic of China

**Keywords:** Case report, Congenital hepatic hemangioma, Pulmonary hypertension, Newborn, Therapeutic embolization

## Abstract

**Background:**

Pulmonary hypertension (PH) in newborns is a rare but serious condition that often requires immediate intervention and quick diagnosis of the correct etiology to prevent mortality. Congenital hepatic hemangioma (CHH) is an example of an extrathoracic etiology of PH.

**Case presentation:**

Herein, we report the case of a newborn with a giant liver hemangioma, who presented with an early onset of PH and was successfully treated with intra-arterial embolization.

**Conclusions:**

This case illustrates the importance of suspicion and prompt evaluation of CHH and related systemic arteriovenous shunts among infants with unexplained PH.

## Background

The etiologies of pulmonary hypertension (PH) are very diverse and include congenital heart disease (CHD), parenchymal lung diseases, pulmonary hypoplasia, congenital diaphragmatic hernia, sepsis, perinatal depression, and in utero medication exposure (selective serotonin reuptake inhibitor and nonsteroidal anti-inflammatory) [1]. Although intrathoracic disease and congenital heart defects are the most common etiologies, extrathoracic causes, and non-cardiac causes must be considered [2]. Congenital hepatic hemangioma (CHH) is an example of a potentially fatal prognosis if not positively managed. Here, we report the case of a newborn who presented with tachypnea and tachycardia. It has been established that the newborn’s symptoms were due to PAH secondary to CHH.

## Case presentation

A 23-year-old primigravida was referred to our hospital at 37 weeks of gestation because the B ultrasound showed that the fetal right atrium was enlarged, the lung-to-host ratio was 0.69, and the hepatic vein was widened. Maternal history was unremarkable, with the routine first- and second-trimester ultrasound scans reported as normal. At 37 + 2 weeks, an elective cesarean section was performed, delivering a female infant weighing 2750 g and with Apgar scores of 9 and 10 at 1 and 5 min, respectively. On the first postnatal day, the physical examination revealed tachypnea and tachycardia. The breath sounds were equal bilaterally. Cardiovascular examination revealed a grade 3/6 holosystolic murmur best heard at the left parasternal second intercostal border, with no thrill. The liver was tangible 3 cm below the right costal margin. No vascular malformations were noted over the skin and no splenomegaly was not observed.

A complete blood count revealed mild anemia. Evaluation of cardiac function revealed markedly elevated (33,198 pg/mL) B-natriuretic peptide (BNP). Liver function tests were all within normal limits apart from decreased total protein (45.9 g/L, normal range: 49–71 g/L) and albumin (30.4 g/L, normal range: 35–50 g/L). There was no evidence of renal function dysfunction (creatinine: 63 μmol/L; blood urea nitrogen: 6.5 mmol/L). Serum α-fetoprotein level (> 3000 ng/ml) was above normal limits for the neonatal period. There was no evidence of hepatitis B or A virus infection.

**Fig. 1:**
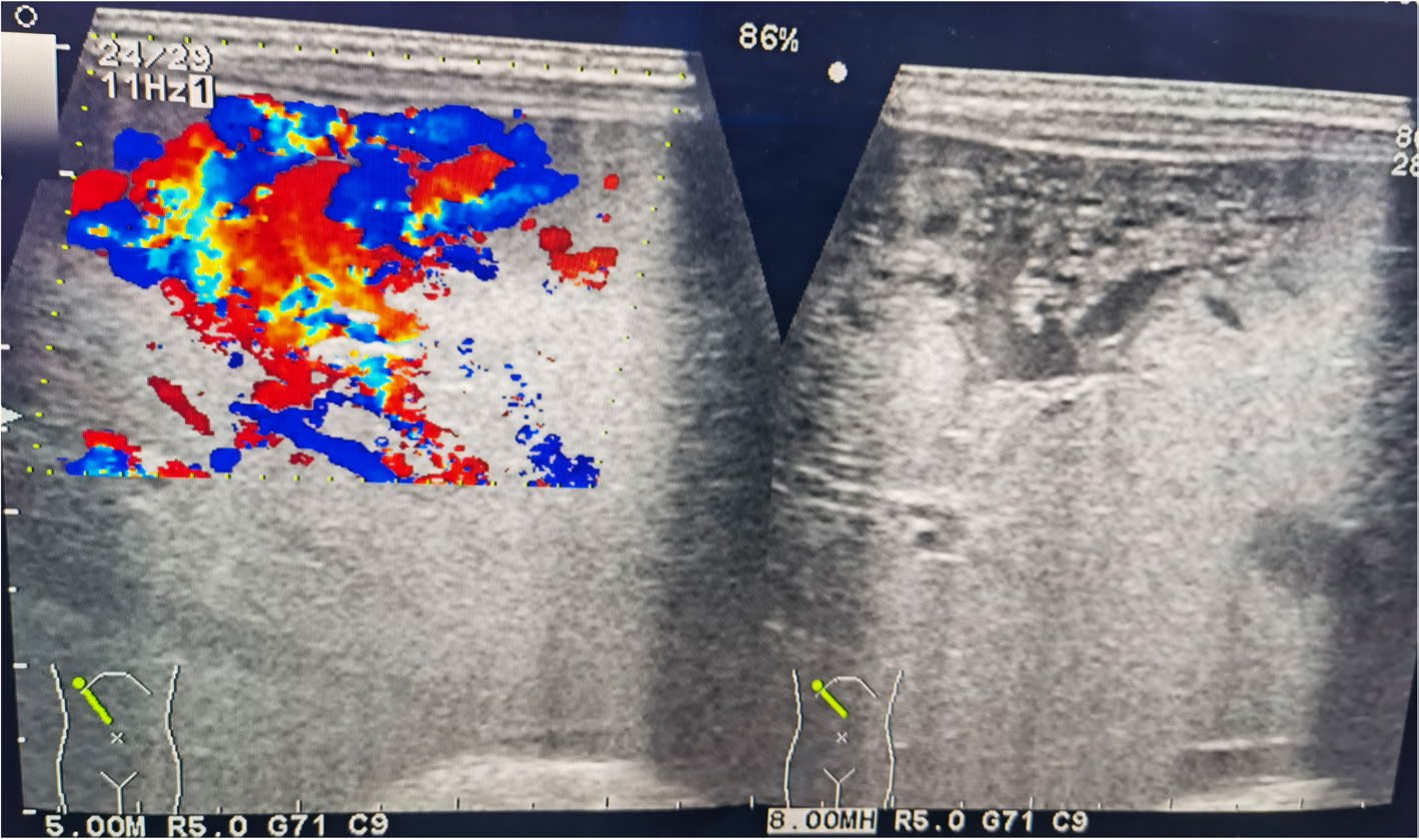
Abdominal ultrasound showed cavernous transformation in the liver with the intralesional massive turbulent vascular flow

We performed echocardiography, which revealed a heart with a maximum tricuspid regurgitation jet velocity of 4.7 m/s (estimated pulmonary artery systolic pressure of 88 mm Hg), atrial septal defect (ASD) 4.9 mm (bidirectional shunt), patent ductus arteriosus (PDA) 3.0 mm (bidirectional shunt), dilated right atrium and right ventricle, indicative of PH. Chest radiograph revealed an enlarged cardiac silhouette with increased pulmonary vascularity without pneumonia. Given the presence of hepatomegaly, an abdominal ultrasound (US) (Fig. 1) was performed, which revealed cavernous transformation in the liver with the intralesional massive turbulent vascular flow. Surprisingly, the portal vein was slightly thickened, and the left, middle, and right hepatic veins were significantly dilated.

**Fig. 2:**
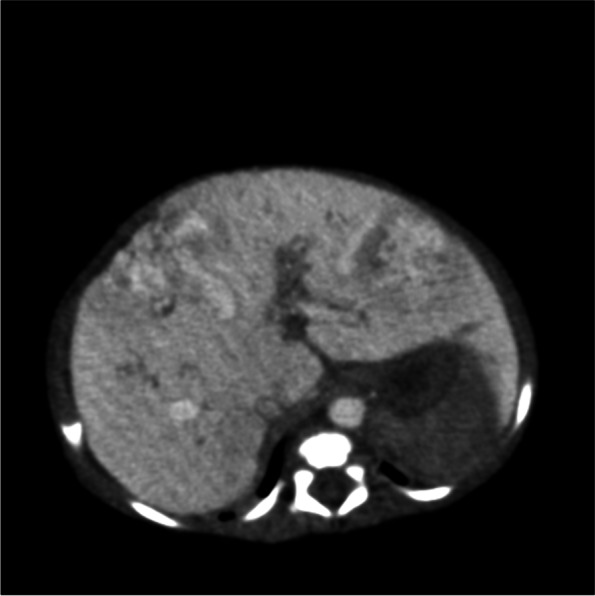
Computed tomography (CT) scans of a patient with Congenital hepatic hemangioma

An abdominal CT scan (Fig. 2) revealed an enlarged liver and multiple patchy hypodense foci in the liver. After contrasting medium injection, the lesions showed an enhanced signal intensity. The right intrathoracic artery was markedly thickened and tortuous, with the end entering the liver. A patchy heterogeneous area of enhancement was seen in the right anterior lobe of the liver and the left lobe of the liver, and the margins of the liver were not shiny, with thickened vessels inside. The veins of the middle lobe of the liver and the left hepatic vein were both noticeably thickened.

**Fig. 3:**
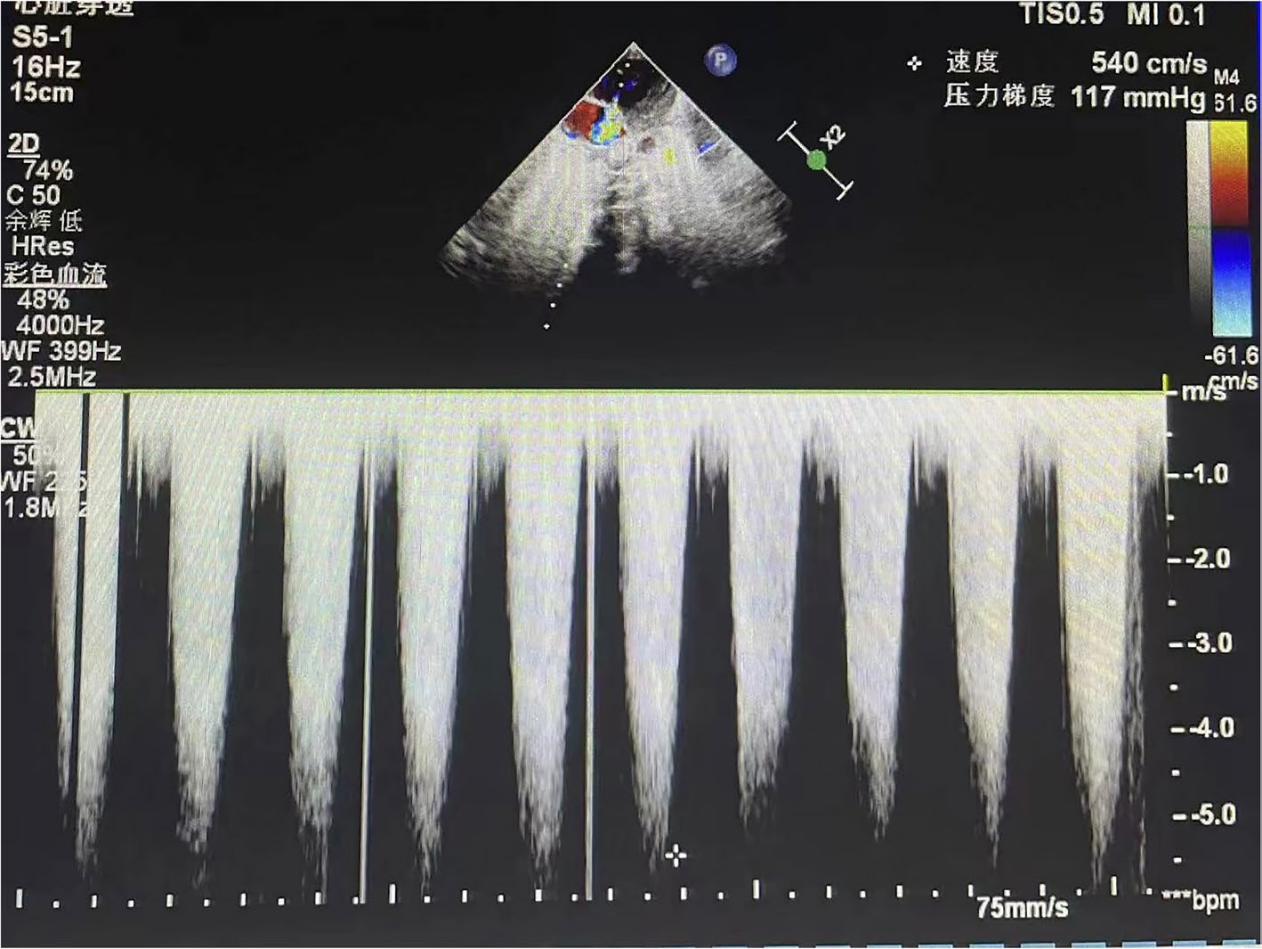
Echocardiogram showed tricuspid regurgitation with a peak jet velocity of 5.4 m/sec

The patient was diagnosed with PH secondary to CHH due to the enhanced pulmonary blood flow from the congenital hepatic vascular shunt. The newborn was treated with diuretics and fluid restriction. Serial echocardiograms were performed to follow the improvement in PH. However, on serial echocardiograms, pulmonary arterial pressure showed an increasing trend (88 mmHg-110 mmHg-117 mmHg) (Fig. 3). At this crucial juncture, the benefits and risks of arterial angiography and embolization were considered, and a decision was made to embolize the major vascular feeders. Informed consent was acquired from the parents with the understanding that the major indications for this procedure were PH and CHH at 20 days after birth. Preoperative preparation included an electrocardiogram, an abrosia diet, and total parenteral nutrition.

**Fig. 4:**
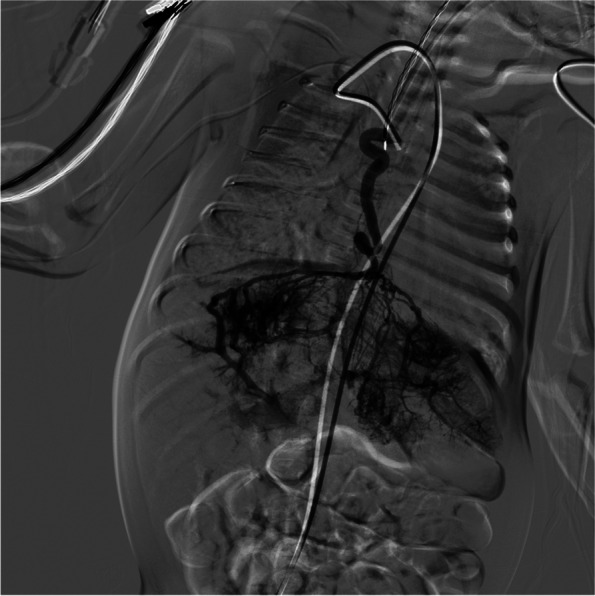
The right internal thoracic artery angiography showed that the right internal thoracic artery was supplying blood to the liver, and multiple vascular clusters were seen in the liver

**Fig. 5:**
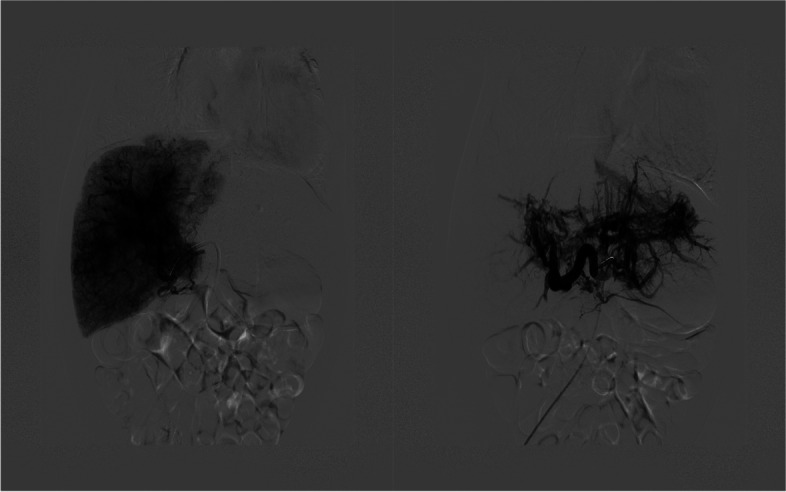
Contrast injection in the hepatic artery

Under general anesthesia, the innominate artery was accessed under ultrasonic guidance, and a 4F elbowed catheter was advanced into the right internal thoracic artery to the anterior bifurcation of the liver tumor vessels. Gelatin sponge particles were slowly from the right internal thoracic artery to the blind end (Fig. 4). A 4F straight flush catheter was advanced into the proper hepatic artery via femoral arterial-celiac trunk access. After angiography, digital subtraction angiography (DSA) was used to guide selective catheterization and gelatin sponge embolization of the right and left hepatic arteries. Branches at all levels were embolized (Fig. 5). The patient was transferred to the neonatal intensive care unit after surgery.

**Fig. 6:**
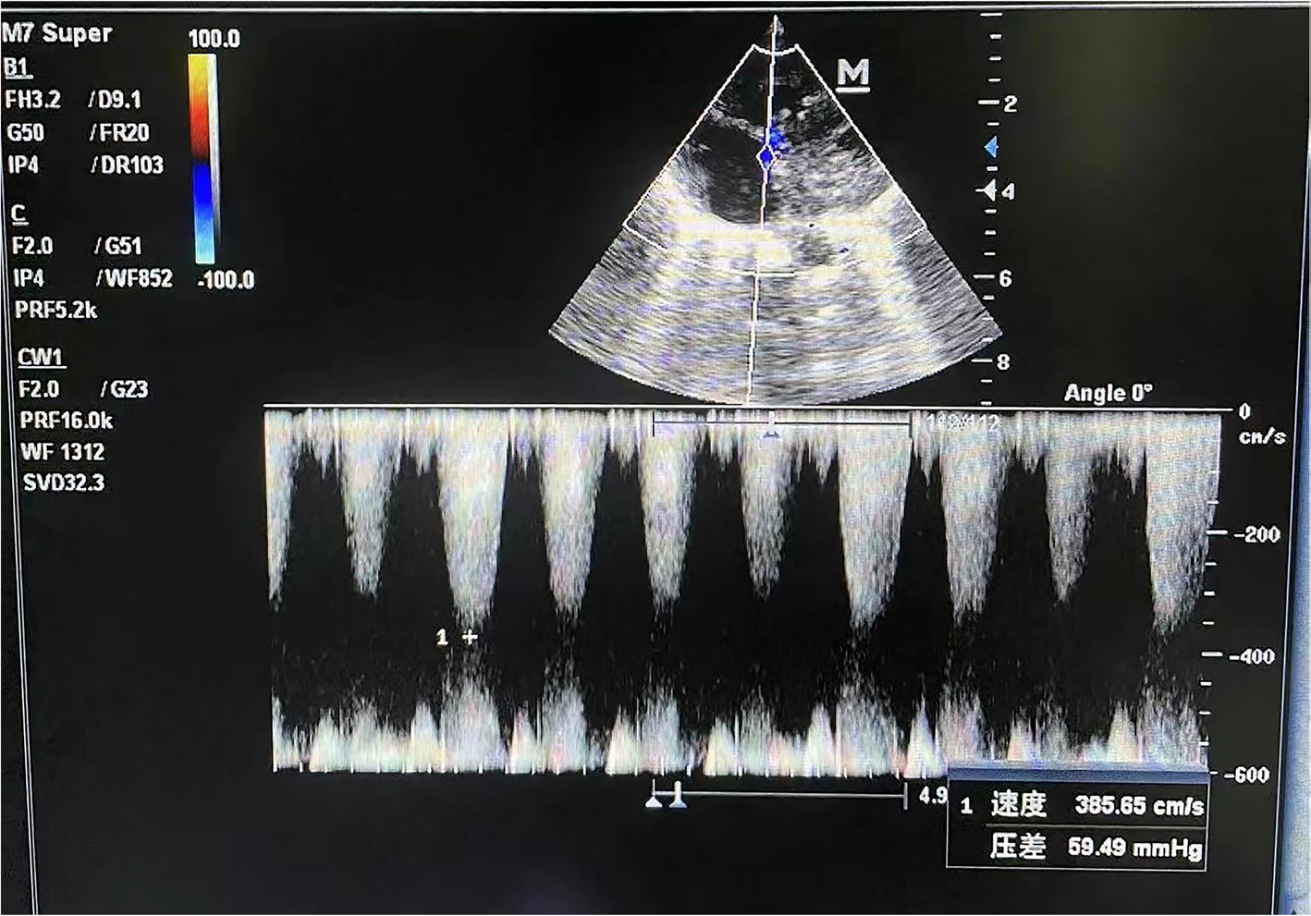
Echocardiogram showed tricuspid regurgitation with a peak jet velocity of 3.9 m/sec

**Fig. 7:**
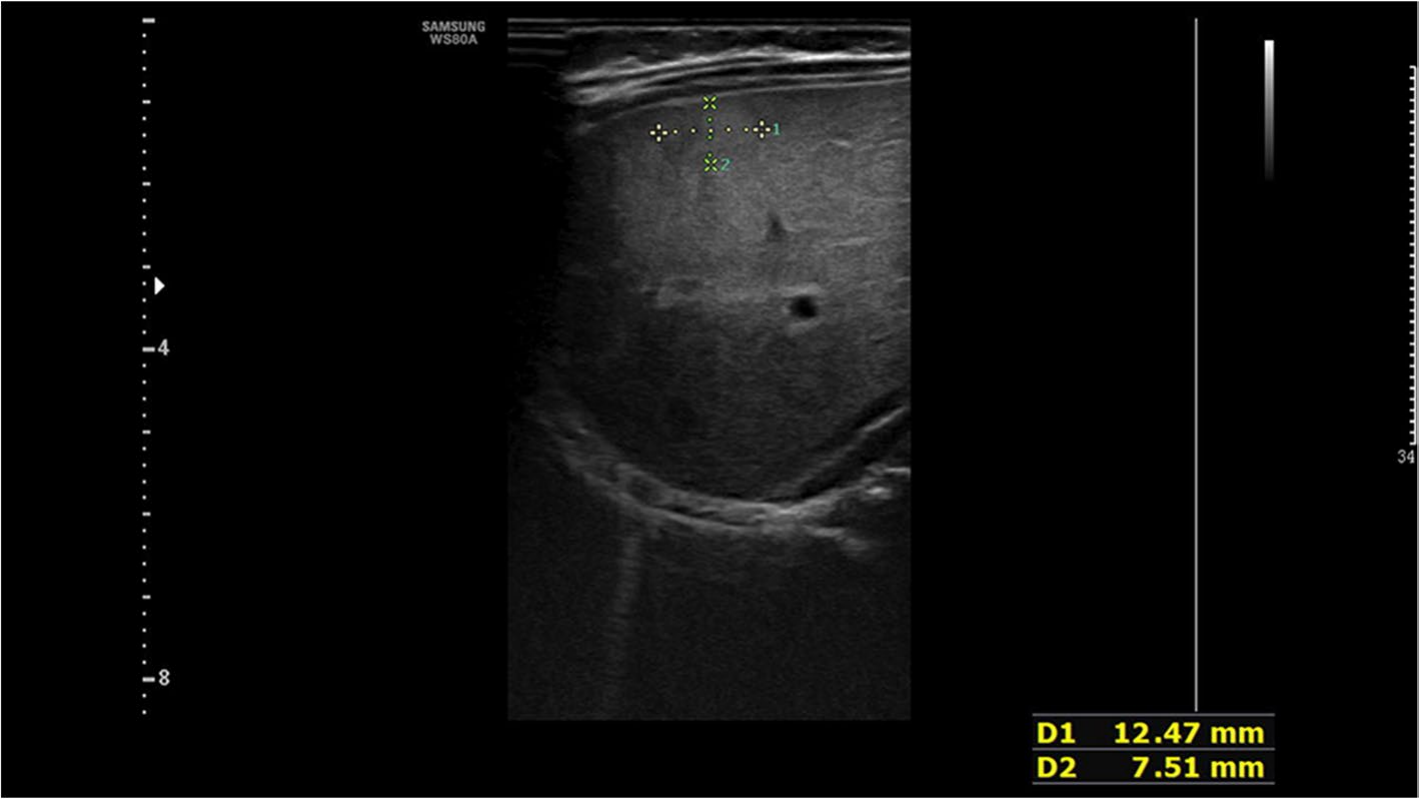
Liver mass 4 months after hospital discharge

During the postoperative recovery stage, the newborn gradually stabilized. Abdominal scans revealed a progressively smaller hepatic mass. On serial echocardiograms, pulmonary arterial pressure showed a decreasing trend with normal pulmonary arterial pressure (117 mmHg-59 mmHg-33 mmHg) (Fig. 6) 3 weeks after surgery. Serum α-fetoprotein levels dropped to normal limits during the neonatal period, and BNP levels showed a significant reduction. To enhance the involution of the CHH, the patient was treated with oral propranolol 3 weeks after surgery. The dose of propranolol was increased to a maximum dose of 0.5 mg/kg/day, once every 3 to 4 days to a maximum dose of 2 mg/kg/day. Post-discharge follow-up visits confirmed a continued response to treatment at the time of writing, and a significant reduction in hepatic mass at 4 months of follow-up (Fig. 7).

## Discussion and conclusion

For the fetus, PH is a normal and necessary state. A dramatic change occurs in the pulmonary circulation at birth, going from a condition of high resistance and low flow in utero to a low resistance and high flow circuit shortly after birth [3]. When normal cardiopulmonary conversion fails to occur, for example (pulmonary vascular injury or delayed diastole), this results in pulmonary hypertension. Many diseases cause pulmonary hypertension. As reported by the current WSPH Pediatric Task Force, associated disorders can be generally characterized as one of three types: intrathoracic disease (infection, asphyxia); structural cardiac diseases, or systemic arteriovenous fistulas [4]. Systemic arteriovenous fistulas such as a cerebral vein of Galen malformation, large cerebral arteriovenous malformations, and hepatic arteriovenous malformations can lead to an obligatory pulmonary over circulation and have been reported to manifest with PH in the immediate postnatal period. Waldo [5] has reported intrauterine findings of a large hepatic hemangioma. After delivery, the infant remained stable without any signs of cardiac insufficiency or other complications. After an uncomplicated neonatal course, the infant was discharged home on the 10th day after birth, while the other case died in utero. Matthew [6] and Zhu both [7] reported on IHH of cardiac output, which was treated with drugs and surgery respectively. Consequently, any neonate presenting with unexplained PH needs to be excluded from multiple arteriovenous fistulas, including hepatic and cranial systemic arteriovenous fistulas, after the cardiopulmonary disease has been excluded. Moreover, appropriate imaging studies should be performed.

The newborn in the case study presented with symptoms and signs in compliance with PH, although she had no severe lung diseases or a structural cardiovascular system. She had a noncardiac cause of PH secondary to the extracardiac shunting from arteriovenous malformations in the liver. Hepatic tumors among pediatric patients are rare, accounting for only 2%—3% of all pediatric solid tumors [8]. Infantile hepatic hemangioma (IHH) is the most common hepatic tumor occurring in infancy. However, CHH is a rare condition. The precise incidence is unknown, but it likely occurs in less than 1% of all infants [9]. The mean age of onset of PH due to congenital arteriovenous malformations in the liver was reported to be 4—5 years of age; nevertheless, both could be discovered during the neonatal and early infantile periods [10]. Furthermore, the shunt vessel size in CHH did not correlate with the emergence of PH [11, 12]. The mortality rate of PH caused by CHH is approximately 40–50% in utero and generally reaches its peak size before or at birth. Newborns may present with thrombocytopenia, hypofibrinogenemia, intratumoral bleeding, and anemia after birth [13]. Meanwhile, CHH can act as an arteriovenous fistula causing life-threatening high-output cardiac failure and respiratory distress. Similarly, our patient had symptoms characteristic of PH, tachypnea, and cardiomegaly after birth. Liver ultrasonography is the most often performed initial diagnostic test for the radiologic diagnosi00s of CHH, but MRI and CT are the modalities of choice for radiologic confirmation of CHH. The patient’s diagnosis was confirmed using a combination of ultrasonography and CT scan (Figs. 1 and 2, respectively).

There are diverse medical treatments for liver hemangiomas, including follow-up observation, medical, endovascular, surgical, and a combination of these therapies. The therapy chosen mostly relies on the clinical condition and the experience of the treatment center. In patients with or no mild symptoms, close follow-up observation may be appropriate. CHH is a high-flow vascular tumor that can cause PH and cardiac failure. Therefore, symptomatic CHH requires aggressive treatment. Surgery has been advocated when follow-up observations and medical therapies are insufficient. Invasive interventions, including surgical resection and embolization, are all available method modalities that can enhance overall prognosis.

In our patient, progressive PH required intervention. Our treatment was initially focused on liquid capacity, with diuresis and fluid restriction as needed. However, with the increasing severity of PH, severe AP, and AV shunts, emergency transcatheter intervention (arterial embolization) was thought to be the appropriate and least-invasive treatment of choice compared with surgery. Successful reduction of the cardiac preload following embolization improved the PH in our patient improved following embolization. There was no obvious complication regarding the embolization procedure. Modern equipment with very fine catheters and new material has made super-selective embolization in newborns possible. However, an expert’s technical skills are required [14]. Based on our encouraging results, embolization can be recommended as the first treatment for symptomatic vascular malformations even in newborns.

We have reported a case of CHH in a term newborn presenting with PH. Due to the significantly increasing PH and dilatation of the heart, an arterial embolization of the liver hemangioma was therapeutic, and she improved quickly after the embolization.

In conclusion, CHH and associated systemic arteriovenous shunts need to be suspected and promptly assessed when newborns present with unexplained pulmonary hypertension. Perinatal management of this kind of condition is a challenging task and should be carried out in tertiary centers with equipment including NICU and endovascular interventional techniques and pediatric surgery.

## Data Availability

All data generated or analyzed during this study are included in this published article.

## Abbreviations

PH: Pulmonary hypertension
CHH: Congenital hepatic hemangioma
CHD: Congenital heart disease
IHH: Infantile hepatic hemangioma
